# Is Community-engaged Learning Possible During a Pandemic: A Call for Culturally Competent Medical Education

**DOI:** 10.15694/mep.2020.000264.1

**Published:** 2020-11-26

**Authors:** Nirmala Prakash, Joel Grunhut, Heather Howard

**Affiliations:** 1 Florida Atlantic University Charles E. Schmidt College of Medicine; 2 Florida Atlantic University Sandler School of Social Work

**Keywords:** education, community, community-engaged learning, cultural competency, COVID-19

## Abstract

This article was migrated. The article was marked as recommended.

Previously, medical school curriculum focused on faculty or physician-led basic science and traditional clinical experiences, with medical students only gaining experience of the community in which they practice during residency. In an effort to enable students to understand US healthcare disparities, the introduction of public health topics regarding marginalized communities and underrepresented individuals have been included in the classroom. However, missing from this shift is the inclusion of authentic public health educational experiences for medical students. These learning experiences are vital to truly understanding the marginalized and discriminated patient populations physicians will encounter. The recent COVID-19 pandemic has brought forth challenges for medical educators in numerous ways including how to effectively prepare students in understanding cultural competency through community-engaged learning for a new set of patient population; the pandemic patient. Due to health disparities, each patient experienced this pandemic differently based on their individual, cultural and community setting; also highlighting the importance of community-engaged learning. Here, the authors posit the role and importance of community-engaged learning in medical education and its utilization during the changing medical landscape due to COVID-19.

## Introduction

Over the last few decades, medical school education has evolved to train students in cultural competency and to address the broader underlying health needs of a population ( Meurer *et al.,* 2011). By concentrating on underlying issues affecting patients, the prevalence of using community-engaged learning to shape culturally competent physicians is increasing in medicine. Cultural competence is a set of congruent behaviors, knowledge, attitudes, and policies that come together in a system, organization, or among professionals that enables effective work and clinical care in cross-cultural situations ( Flores, 2000). Teaching cultural competence is a challenge because now medical educators must provide their students knowledge of the differences amongst patient populations ( DasGupta *et al.,* 2006).

## A Didactic Lecture Limits the Expansion of Medical Training

Traditional didactic medical education does not capture the nuanced perspectives and authentic voices needed when teaching about social determinants of health (SDOH) topics such as violence, substance use disorder and racial disparities. This traditional approach cannot capture the true influence of lifestyle, socioeconomic factors, and environmental hazards on morbidity, mortality, and well-being ( Betancourt *et al.,* 2002). A single lecturer is limited in his/her cultural knowledge of a given different culture or community. Medicine is a social practice that greatly depends on how patients are categorized. However, these categorizations are often implicitly filled with bias and prejudice ( Meurer *et al*., 2011). These stereotypes lead to false judgements and sometimes lead to adverse outcomes through oversimplification of physiology and pathology ( Meurer *et al.,* 2011). In the hospital setting, these prejudices translate to wide ethnic and social disparities in diagnosis and treatment (Nelson, 2002). Additionally, a lecture format does not sufficiently impact a medical student’s aim to have a deeper understanding of complex issues ( DasGupta *et al.,* 2006). A lecture may provide adequate and useful information for outlining social issues but it does not create a profound change of understanding for those listening, teaching methods beyond discussion are required ( Kamaka, 2010). Teaching culturally competent care is complex and requires a multiple levels of learning to uncover prejudices and implicit biases ( Kamaka, 2010). Recently, COVID-19 brought to the forefront health disparities. For example, Racial and ethnic minorities, immigrants, LGBTQ, women and individuals from other marginalized groups have routinely fared worse in health outcomes as compared to their White counterparts (Adams *et al.*; Cleveland Manchanda *et al.,* 2020). COVID-19 death rates varied amongst diverse populations magnifying inequalities in health and the effects of SDOH on life span, access to care and culturally competent health care. A thorough and thought-provoking teaching method is necessary to cover these issues.

## The Role of Community-Engaged Learning

In an effort to expand the educational and experiential opportunities for students and faculty alike, educators have begun implementing novel learning experiences. Community-engaged learning creates opportunities for first-hand and lived experiences with diverse populations and community partners who provide front-line care and program delivery. These approaches have seen success at the few medical schools beginning to implement these ideas into practice. Medical schools have reported these experiences as key to their students deeper understanding of the social intricacies in health disparities ( Roughead *et al.,* 2017). Students are given a greater sensitivity to communities they engage with and learn about the specific circumstances making these populations so vulnerable ( Ventres & Dharamsi, 2015; Ventres *et al.,* 2018). Additionally, students are able to develop the ability to foster relationships with community leaders and individual community members. A systematic review analyzed 57 peer-reviewed articles regarding methods of community based learning which demonstrated that educators found it easier to teach these complex and sensitive topics through community engagement more than didactic lectures ( Boroumand *et al.,* 2020). Introducing students to the community earlier in a medical student’s education creates opportunities for them to see and practice interactions with marginalized communities. This practice can help to develop the humanistic skills necessary to effectively care for all populations sooner and can create personal practices integrating social determinants of health for the future clinical years.

## Culturally competent community-engaged curriculum

Medical school curricula across the US may benefit on developing curriculum in health inequities and social determinants of health with a similar innovative community-based approach. SDOH topics such as LGBTQ health, stigmatized patients, refugee health, human trafficking, child sexual abuse and gun violence may benefit from a community-engaged learning approach. Community-engaged learning techniques such as community led panels, early preceptorship, standardized patient scenarios, and service-learning, to name a few, add veracity and credibility to medical education learning objectives that aim to address cultural competency and social determinants of health. A community-based medical school less than 10 years old with less than 100 students per class developed a culturally sensitive curriculum with hands-on training in how to be responsive to the communities students encounter. The following are examples of strategies used at a new, small medical school to implement the community-engaged curriculum.

## Community led panels

The medical school educators reimagined the classroom barriers by inviting service and community partners in and bringing students out of their classrooms. Community partners who focus on social issues provide the best experiential knowledge and teaching methods to give students an active role in learning. For example, teaching about sexual violence and human trafficking becomes authentic, timely and relevant when leaders from a local Human Trafficking Task Force, law enforcement and Victim Services produce and present lectures, panels and small group exercises for medical students. This creates an encompassing multi-faceted learning experience on the social issues physicians will potentially encounter in practice. Other panels include LGBT peer panels that provide students with socially relatable peers to better understand the LGBT community. Additionally, the medical students experienced true interprofessional education by interacting with students and educators from school of social work and school of nursing in patient scenarios. These processes allow effective integration of health advocacy and cultural competency into medical education ( Seifer, 1998).

## Early student preceptorship

Students can gain insight with exposure to patient scenarios by beginning preceptorship in their first year. Although some may argue this is too early in a student’s medical education, there is a vital component of learning about social determinants of health to be gained. Students are likely to have enough basic clinical training halfway through their first year to be adequately prepared for preceptorship, and through continuous actual patient interaction students will pick up the underlying social health issues patients face ( Rooks *et al*., 2001; Henschen *et al.,* 2020). Additionally, rotating first year students through non-for profit clinics once to twice a semester is an invaluable experience for learning socioeconomic, racial, and language barriers. These rich experiences support student growth in cultural competence and understanding in addressing the social determinants of health.

## Authentic voice via standardized patients

Additional thoughtful learning experiences can be created through a curriculum designed with standardized patients that display social health issues such as substance use disorders in addition to the clinical lesson being taught ( Rutledge *et al.,* 2004). Creating authentic standardized patient experiences using individuals from the community and ensuring racial, gender, sexual orientation, disability are represented, also contribute to a community-engaged learning experience and development of cultural competence. A patient representing a marginalized group can help the student become more aware of implicit biases in practice. Participation in a post encounter breakout session discussing these issues creates reflective and critical dialogue amongst students and faculty.

## Learning Opportunities in a COVID-19 World

COVID-19 has left physicians with many questions about the affected patient populations and what are physicians roles in addressing social inequity. We know that major Black communities had a triple infection rate and an astonishing six times mortality of major White communities ( Kendi, 2020). At this point in time we do not fully understand why this population was impacted more. Perhaps the biggest issue amongst clinicians is can we change the outcomes in the event of another pandemic? Can these patient populations be better equipped to handle a similar outbreak? The COVID-19 crisis forced physicians to make difficult decisions that may have been better guided with more information and better understanding of their patients. Now more than ever, during the aftermath of COVID-19, medical students must engage with their communities to understand the social determinants of COVID-19 and to gain insight into how the pandemic affected the lives of their future patients. Likewise, an additional call for community-engaged learning post COVID-19 stems from an unprecedented rise in US unemployment that will inevitably translate to a rise in economically challenged populations ( Blustein *et al.,* 2020). Healthcare is often compromised in disadvantaged individuals because of the unavoidable time and monetary constraints ( Woolhandler & Himmelstein, 2020). Students can receive great learning experiences during these changing times by interacting with this expanding socioeconomic group.

## Call for community-engaged learning post COVID-19

Until COVID-19, all community-engaged learning was performed face-to-face. Now educators are faced with a question of what is the role of community-engaged learning in a world that may need to enforce social distancing and other protective precautions? Exposure to these communities is essential so medical students can uncover and learn what barriers individuals faced, and the type of care they received. Students will learn Black communities often have less ability to social distance because of crowded housing, have more exposure to pollution, and have different baseline levels of chronic diseases (Adams *et al*.; Cleveland Manchanda *et al.,* 2020). These disadvantages put them at higher risk of contracting and dying from COVID-19 (Adams et al.; Cleveland Manchanda *et al.,* 2020). Observing and interacting in some form with community engagement should not be compromised.

## Interacting remotely with community led panel

Understandably, many institutions will limit student physical interactions but medical education faculty may need to provide alternative methods of community-engaged learning. One strategy to understand the issues that face minority patient populations is to include in the curriculum a learning session on COVID-19 that invites community partners to join a virtual dialogue about how to better respond to a pandemic. Engaging one and one remotely or with socially distanced group site visits, students will find common shared experiences and note the differences between how individuals dealt with the pandemic. Similarly, panels with members of interprofessional education or combined panels to help better understand substance use disorder can transition to remote platforms. For example, the authors recently invited recovery and peer support specialists to co-facilitate a virtual session with internal medicine residents discussing implicit and explicit bias and how to engage with marginalized patients via an online virtual meeting. Cultural immersion is still feasible with remote platforms. Additionally, video conferencing will allow interprofessional and multi-institution involvement to occur with ease. It is vital to understand that even in remote learning formats, medical educators can use technology to maintain and deepen community-engaged learning. This can be achieved through remote learning platforms that invite community members to small group discussions to expose students to others’ challenges during COVID-19. Learning firsthand will give medical students an enormous advantage in understanding the intricacies of different populations and how they were affected through COVID-19.

## Telehealth and preceptorship

During COVID-19, the landscape of medicine changed and it was accelerated by technology. Telemedicine showed significant positive aspects during COVID-19 and it can be expected that it will have an important role in the future and health education as well (Hollander & Carr 2020). Telehealth has unique features to offer to enhance medical education and help students. Telehealth training utilizing standardized patients that represent lower socioeconomic patients will also help students gain appreciation for a patient’s full story. Preceptors using telehealth and school affiliated hospitals should both begin to conceive methods of implementing telehealth into their medical education role. The use of telehealth is helpful in having immediate access to patients and for ease of follow-up care. These same reasons apply for medical students wishing to follow up with patients. Preceptors and hospitals can obtain a rigorous learning environment through installation of a webcam in a patient’s room that students can view and contribute by obtaining patient history of present illness and adding to the physician-patient interaction. The role of this technology in rotations and clerkships may have a basis depending on results of this utilization by first year medical students.

## Remote service project learning

In a situation where social distancing must be implemented medical students can still lead service learning projects to help expose themselves to other communities. For example, students can join food pantries where social distancing is maintained and as an additional service they can educate those in line in standard hygiene precautions. Students can speak to those individuals to better understand the barriers to healthcare for the recently unemployed. In medical schools across the country students have begun to volunteer in call centers, helped create patient education material, and helped with grocery shopping among other activities ( Rose, 2020). Even grocery shopping may enhance the student’s education through student guidance to clients with medically informed nutrition input and increase cultural competence.

Additionally, technology can aid students in performing service learning. Mentoring economically challenged high school and college students, meeting with elderly populations, and helping disabled children with their schoolwork can all be done virtually as well and will also provide a better idea of the community the medical student works with. These methods increase student engagement and understanding of underrepresented and marginalized people while providing humanistic and compassionate care.

## Discussion

There are various approaches to enhance a curriculum that encompasses community-engaged learning. Doing so is vital in educating future physicians because these experiences bring forth the many aspects of patient issues that are difficult to teach strictly by didactic approaches. These experiences can be shared through community led panels, service learning projects and minority cases for standardized patients. Although there are some limitations to community engagement because of COVID-19 such as social distancing requirements, community-engaged learning must not be totally dismissed. Students and faculty may address these obstacles through the use of technology and socially distanced interactions. Community led panels, early preceptorship, novel service learning projects and minority cases for standardized patient methods can all be done virtually as well, further raising their importance as a mode of learning during a post COVID-19 world. With the current pandemic, future medical education research should focus on advancing methods for community-engaged learning. Medical schools must continue to be global leaders in education and community care during these new and changing times by augmenting their curriculum to satisfy this need.

## Take Home Messages

• Community-engaged learning is a vital component of medical education
• Students can participate in community led panels, service learning projects and minority cases for standardized patients
• COVID-19 has brought forth the importance of community-engaged learning.
• Following COVID-19, educators should implement novel community-engaged learning methods

## Notes On Contributors

**Nirmala Prakash**, PhD, is an Assistant Professor of Integrated Medical Science, Director for Diversity and Inclusion, Florida Atlantic University Charles E. Schmidt College of Medicine, Boca Raton, Florida.

**Joel Grunhut** is a M2, Florida Atlantic University Charles E. Schmidt College of Medicine, Boca Raton, Florida. ORCID ID: https://orcid.org/0000-0002-8104-9679.

**Heather Howard**, PhD, is an Assistant Professor at the Florida Atlantic University Sandler School of Social Work, Boca Raton, Florida.
