# Supplementary material for: Is Community-engaged Learning Possible During a Pandemic: A Call for Culturally Competent Medical Education
Source: MedEdPublish (2016). 2020 Nov 26;9:264. [Version 1] doi: 10.15694/mep.2020.000264.1 (PMC10697631; doi:10.15694/mep.2020.000264.1)
Supplement: Peer review history

# Reviewer report: Reviewer response for version 1

**DOI:** 10.21956/mep.20201.r31268

**Published:** 2021-04-09

**Authors:** Subha Ramani

**Affiliations:** 1 Harvard Medical School, Brigham and Women's Hospital

This review has been migrated. The reviewer awarded 4 stars out of 5 I enjoyed reading this paper on the importance of engaging medical students in cultural competency curricula through community engagement. I agree with the authors that authentic learning experiences are important early on in health professions training. Cultural competence is clearly defined, its importance well emphasized and overall the paper is well written.I agree with my fellow reviewer on the following points:- The abstract could be tighter and expand on the most important messages- This definitely reads like a perspective or reflective piece- nothing wrong with that, but it needs to be clear- If facts or numbers are quoted, readers need to know if they are based on studies or authors' opinions. Again, opinions are fine in a reflective or perspective paper- just need to clarify.- MEP caters to a worldwide audience, terms should not be country centric and facts and figures should be relevant to the rest of the worldI would have liked to have seen a road map to where the authors are taking me at the end of the introduction section.This is an important topic, the pandemic indeed has uncovered disparities and I commend the authors for bringing attention to the topic.All health professions educators should find this piece interesting and it will provide food for reflection.

Reviewer Expertise:

NA

No decision status is available

# Reviewer report: Reviewer response for version 1

**DOI:** 10.21956/mep.20201.r31267

**Published:** 2021-02-10

**Authors:** Ken Masters

**Affiliations:** 1 Sultan Qaboos University

This review has been migrated. The reviewer awarded 3 stars out of 5 An interesting paper dealing with community-engaged learning during a pandemic.Community-engaged learning is crucial to medical professional training, and, as the authors note, the issues arising from the pandemic have exacerbated this need. The paper is a useful read, giving interesting aspects for medical educators to consider. It does, though, have some issues that should be addressed:• The Abstract gives a good background to the paper, but the main ideas given in the paper are then squashed into a single sentence or two at the end of the Abstract. It might be better to shorten the first part of the Abstract and broaden the second part, so that the reader can get a better understanding of what is in the paper. (The Abstract should be a summary of the main points in the paper, rather than a background lead-in).• Each section consists of a single paragraph, and the reader is met with a wall of text. It would be easier for the reader if these paragraphs were broken into smaller pieces for easier understanding.• The authors need to write out the LGBTQ acronym, particularly because they sometimes use LGBTQ and sometimes LGBT. They may wish to explain the difference. As readers who would benefit most from the paper would be those unfamiliar with the terminology and issues, these need to be clear, otherwise, the authors’ efforts may be wasted. • The information about “A community-based medical school less than 10 years” is too vague. If the authors are citing a study, then it needs to be formally cited and referenced. If the authors are giving their own original results of research, then they need to supply more information about the work. In either case, the institution needs to be identified.• “The following are examples of strategies used at a new….” No further examples are given in this section. It would appear that the examples are given in the next section (but not the other sections that follow). The authors should clarify this, so that it is clear where the examples they cite from that study begin and end.• It would be useful if the authors could give some details about the context in which they are working, and the background experience of their students; medical curricula are different around the world, and a sentence like “Students are likely to have enough basic clinical training halfway through their first year to be adequately prepared for preceptorship” would not make sense in many countries.• The statement regarding the mortality rates citing Kendi may need reviewing, for two reasons:o The Kendi article is a magazine article written by a non-medical (albeit academically respected) person; it would be better to cite the original publications on which those figures are based.o From my rather quick reading of the mortality rates, the rates appear to 2 to 3 times the expected rates, based on population sizes. Still unexpectedly high, but not the 6 times given by the statement. If the authors have arrived at their figure by performing extra calculations and extrapolations, then it would be useful if they could lead the authors through that process (but again, if they draw their data directly from the primary sources, it would more strongly support their argument).• I’m not entirely sure that the heading “Discussion” is the appropriate heading for the final section of the paper. Perhaps “Reflection” or something similar might be better.• Similarly, I see that the authors have submitted their paper under a “New education method or tool”. This may leave readers a little disappointed, as many would have been waiting to find out about a new method or tool, but it does not materialise. I think the paper is a good reflective opinion piece, and should be listed as such. (While they do refer to some tools not widely used before, such as telemedicine or community involvement (even in the early years of medical training), these are not new: the important aspect of the paper is the need to apply these during the pandemic. If the authors really wish to keep it as a “New education method or tool” then they would need to clearly highlight and identify the new aspects to what they are proposing, perhaps with practical examples..So, the paper is interesting, but does need a little more work. I look forward to Version 2 in which these issues are addressed.

Reviewer Expertise:

NA

No decision status is available

# Reviewer report: Reviewer response for version 1

**DOI:** 10.21956/mep.20201.r31266

**Published:** 2020-11-26

**Authors:** Maria de los Angeles Fernandez-Altuna

**Affiliations:** 1 Universidad Nacional Autonoma de Mexico Facultad de Medicina

This review has been migrated. The reviewer awarded 2 stars out of 5 Thank you for writting this article from the point of view of cultural competence and educational practice during the COVID-19 pandemic.All medical educators have been faced to a real challenge for community learning and practice. during this pandemic I liked very much the broad approach taking into account not only community based practice, but this practice oriented to cultural competence. Now that the world is struggling with COVID-19, this culturally medical education approach seem plausible and very suitable.In order to improve the manuscript, I would respectfully advise to create a table to resume main features ofeach approach, advantages, etc.I would also advise to include a table with more practical examples which would guide other medical schools to implement your approach.And finally, to say more about how this approach could be effectively incorporated to he curriculum.

Reviewer Expertise:

NA

No decision status is available
